# Tc-99m MDP Bone SPECT/CT Findings of a Patient Detected with a New Mutation in LEMD3 Gene: A Case of Osteopoikilosis

**DOI:** 10.4274/mirt.25743

**Published:** 2018-02-01

**Authors:** Güler Silov, Zeynep Erdoğan, Murat Erdoğan, Ayşegül Özdal, Hümeyra Gençer, Tayfun Akalın, Seyhan Karaçavuş

**Affiliations:** 1 University of Health Sciences, Kayseri Training and Research Hospital, Clinic of Nuclear Medicine, Kayseri, Turkey; 2 University of Health Sciences, Kayseri Training and Research Hospital, Clinic of Molecular Biology and Genetics, Kayseri, Turkey; 3 University of Health Sciences, Kayseri Training and Research Hospital, Clinic of Molecular Biology and Genetics, Kayseri, Turkey

**Keywords:** LEMD3 gene, osteopoikilosis, Tc-99m, MDP, SPECT/CT

## Abstract

Osteopoikilosis is an inherited condition with autosomal dominant trait resulting in sclerotic foci throughout the skeleton. It has been suggested that loss-of-function mutations of LEMD3 gene located on 12q14.3 result in osetopoikilosis. A bp heterozygote deletion was detected in our patient at the cytosine nucleotide at position 1105 with molecular genetic analysis. Although this mutation has not been previously described, it was considered to be the most likely cause of the disease in our patient due to frame shift and premature stop codon formation. As in our case, three phase bone scintigraphy and whole body imaging did not reflect the true extent of lesion sites and lesion activity. SPECT/CT images could reflect lesion location and activity more accurately, and could be a good alternative for differential diagnosis of unexplained bone pain and sclerotic lesions in one examination.

## Abstract (tr)

Osteopoikiloz kemik doku boyunca kemik adacıkları ile karakterize, otozomal dominant geçiş gösteren herediter bir hastalıktır. Mevcut literatürde, 12q14.3 üzerinde bulunan LEMD3 genindeki mutasyonların sebep olduğu gösterilmiştir. Hastamızda yapılan moleküler genetik analizde ‘Yeni Nesil Dizi Analizi’ 1105. pozisyondaki sitozin nükleotidinde bir bp’lik heterozigot delesyon tespit edilmiştir. Bu mutasyon, daha önceden literatürde tanımlanmamış olmakla birlikte; çerçeve kaymasına neden olması ve erken stop kodon oluşturması sebebiyle yüksek olasılıkla hastalık nedeni olarak değerlendirilmiştir. Ancak üç fazlı kemik sintigrafisi ve tüm vücut taraması, bu olguda olduğu gibi gerçek lezyon sayısı ve lezyon aktivitesini yansıtmamaktadır. SPECT/BT görüntüleme bu açıdan iyi bir seçim olarak görülmektedir.

## Figures and Tables

**Figure 1:**
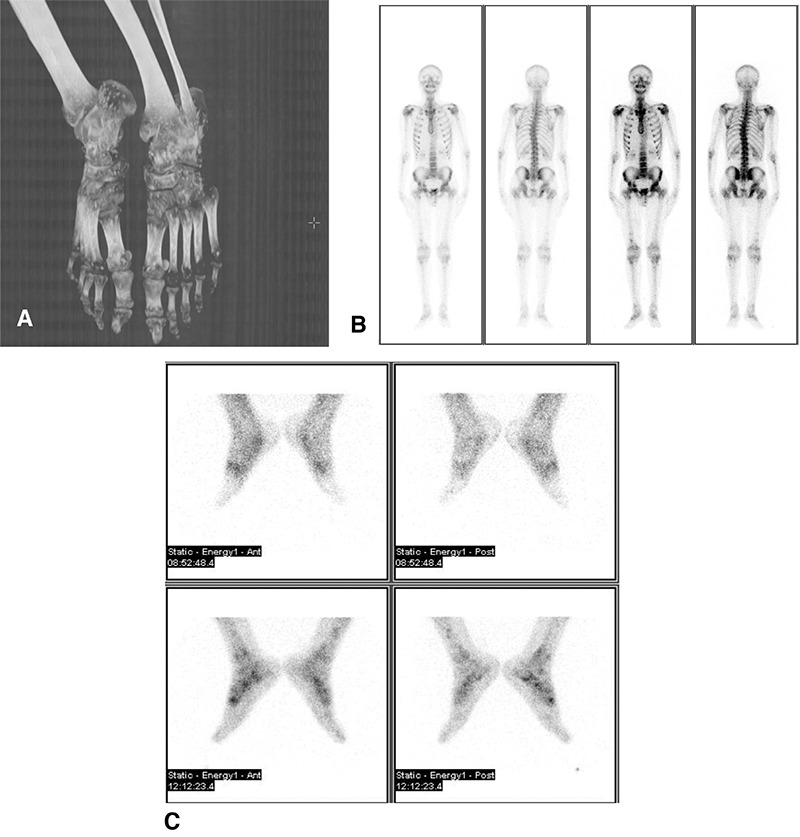
A 20-year-old man was referred to our department with right foot pain. The plain X-ray showed numerous small lesions distributed along the feet (A). A three phase Tc-99m methylene diphosphonate (MDP) bone scan of the foot, whole body scanning along with feet and pelvic-thoracic SPECT/CT were performed. Whole body images showed relative irregularly increased focal MDP uptake on the long bones and pelvic region (B). The late phase static images of feet irregularly increased focal MDP uptake (C).

**Figure 2:**
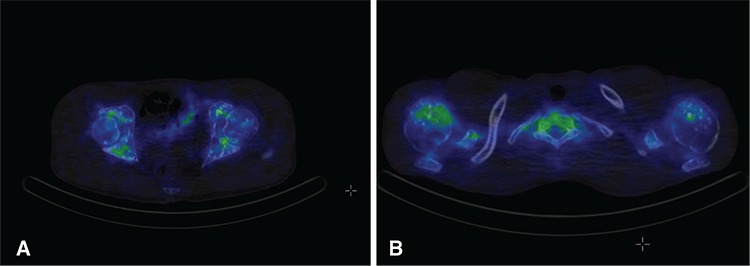
Multiple, sclerotic lesions were detected in thoracic bones, shoulders, and both femur and pelvic bones by CT but radiotracer uptake was observed in some sclerotic lesions on SPECT-CT images (A, B). Laboratory tests were within normal limits except a slightly elevated alkaline phosphatase value of 126 U/L (range: 30-120 U/L). Osteopoikilosis is an inherited condition with autosomal dominant trait that results in numerous sclerotic foci throughout the skeleton (1). Previous studies have reported that these lesions are symmetric but randomly distributed, with increased concentrations at the carpal and tarsal bones, as well as at the ends of the long bones (2,3). Lesions are reported to be located less in the skull, rib, vertebra, and mandible (1). Recently, whole-genome linkage analysis of affected individuals resulted in identification of a loss-of-function mutation in gene LEMD3 at position 12q14.3. LEMD3 is believed to function in bone morphogenetic protein (BMP) signaling by interacting with the family of SMAD proteins downstream from transforming growth factor-beta (TGF-beta) to regulate bone formation (4,5). It has been reported that LEMD3 can antagonize both BMP and TGF-beta signaling in human cells (6). Molecular genetic analysis was performed in our patient, and a bp heterozygote deletion was detected in the cytosine nucleotide at position 1105. This mutation has not been previously described in the literature, but is considered to be the most likely cause of the disease due to frame shift and premature stop codon formation. Histologically, the sclerotic foci correspond to old and inactive remodeling of spongiform lamellar trabeculae with a gross nodular or star-like appearance (7). Sclerotic lesions are classically inactive on whole body scintigraphy (8,9). For this reason, a Tc-99m bone scan is sufficiently diagnostic and usually returns to normal in cases of osteopoikilosis. There have been a few cases reported in the literature of abnormal bone scans in patients with osteopoikilosis (10). In such cases, the pattern of increased uptake is usually symmetric and localized to the distal ends of tubular, carpal, and tarsal bones. This may correspond to active bone remodeling of these multiple foci, which has also been reported in pathologic specimens of osteopoikilosis. As in our patient, localized three phase bone scintigraphy and whole body scanning did not reflect the true extent of lesion site and activity. However, SPECT/CT images could reflect lesion location and activity more accurately, and could be a good alternative for differential diagnosis of unexplained bone pain and sclerotic lesions in one examination.
